# Topics searched by first-time Indonesian fathers during pregnancy journey: An exploratory study

**DOI:** 10.1371/journal.pone.0307051

**Published:** 2024-07-26

**Authors:** Kidung Ageng, Anushia Inthiran

**Affiliations:** Department of Accounting and Information Systems, University of Canterbury, Canterbury, New Zealand; Sefako Makgatho Health Sciences University, SOUTH AFRICA

## Abstract

This study explores the topics searched by first-time Indonesian fathers during the pregnancy journey. Data were collected through semi-structured interviews with a representative sample of first-time expectant fathers in Indonesia. Thematic analysis was employed to identify common themes and patterns in fathers’ search topics and reasons behind the searches. The results reveal that fathers predominantly focus on finding topics related to their partners’ well-being with particular emphasis on health-related topics. Interestingly, the study highlights a lower engagement with topics related to cultural practices, traditions, and religion among first-time Indonesian fathers. Additionally, understanding the situation and finding the solutions to a problem is one of the main popular reasons for first-time fathers to seek a particular topic related to pregnancy. This study provides valuable insights into the topics searched and motivations of first-time Indonesian fathers during the pregnancy journey which have similarities and differences to fathers’ practices in developed countries. The findings underscore the necessity for tailored intervention programs to promote paternal involvement during this transformative period which addresses the unique information needs of Indonesian fathers.

## Introduction

In the landscape of parenting, the journey to fatherhood has garnered increased attention [1–3]. This challenges traditional perspectives that predominantly focus on mothers as the primary seekers and bearers of knowledge in the realm of pregnancy [4]. Furthermore, it also acknowledges the integral contributions of fathers in navigating this significant life transition [5]. Therefore, this underscores the active role of fathers in matters concerning pregnancy. A crucial aspect of paternal involvement during pregnancy is the active pursuit of information related to the various facets of this transformative journey [3, 6]. The knowledge acquired through this process serves as a valuable resource, offering emotional and physical support to both fathers and mothers as they navigate the intricate landscape of pregnancy [7–9].

There are various reasons as to why, first-time expectant fathers perform a search. For example, fathers search to find answers on how to fulfil their role as good fathers [7]. This perhaps is because they feel that the role of the expectant father is not obvious which leads them to validate their assumption of the father’s role in pregnancy. Similarly, most fathers in developed countries explore pregnancy-related topics to understand the roles adopted by fathers and the barriers they may encounter during pregnancy and labour [10]. Additionally, fathers engage in seeking information about pregnancy as a strategy to actively participate in, understand, and adapt to pregnancy behaviours [11].

Fathers in developed countries seek information on various topics related to pregnancy depending on the stage of the pregnancy journey [12]. This behaviour aligns with Wilson’s [13] findings, which suggest information sought is context-dependent. In the first trimester, fathers typically seek information about their partner and the developmental milestones of the unborn child [14]. They also begin looking for healthcare facilities and experts for antenatal care [15]. As the pregnancy progresses, expectant fathers extend their information-seeking to include topics such as childbirth, healthcare, pain management, supporting their partner during and after childbirth, and addressing emotional-related issues [6]. In the period before childbirth, fathers switched from seeking information about pregnancy matters to childbirth [16]. This includes the preparation needed, birthing moment recognition, and childbirth facilities [17]. The information about pregnancy gathered during different stages of pregnancy will be utilised by fathers for future decision-making [18]. The topics searched may differ based on pregnancy stages due to the evolving needs and concerns of expectant fathers as the pregnancy progresses. Therefore, fathers need to adjust their topics to keep engaging with the pregnancy journey and continue supporting their partners.

The type of information sought not only varies based on pregnancy stages but also depends on the geographical location of fathers. Studies indicate differences in the information sought based on geographic location. For instance, fathers in Canada seek information about a healthy diet, personal stress levels, couple relationships, and baby care [8]. In the United States, fathers express interest in nutritional information, supporting their partners, and financial preparation [19], assisting their partners during pregnancy, detecting signs of abnormal pregnancy, and providing support during childbirth [20]. In Sweden, fathers tend to focus more on subjects like painless delivery, healthcare information, postnatal care, and emotional support [21]. Besides preparing for childbirth, fathers in the United Kingdom also prioritise seeking information related to their mental health and well-being in the face of pregnancy issues [17]. On the other hand, fathers in South Nigeria search for information related to pregnancy costs, health insurance coverage and healthcare facilities [2]. This shows that the geographical location might influence the type of information sought by fathers during pregnancy. This is perhaps due to each location’s unique context that shapes the priorities and information needs of the information seekers [22]. For example, specific factors related to geographic location such as access to healthcare services, socioeconomic level, language preferences, and local cultural norms might play a role in shaping fathers’ information-seeking behaviour.

While some information is available on why fathers in developed countries perform a pregnancy-related search [7, 10, 11], there is a notable lack of information on the reasons for first-time fathers searching for pregnancy-related topics in Southeast Asia, particularly Indonesia. There have been some studies found that examining fathers’ exploration of parenting topics in Southeast Asia countries [15, 23–25]. However, this means searching is performed after a child is born. The majority of research around topics searched during pregnancy centred on fathers residing in developed countries [12, 19, 20]. These studies may enhance understanding of fathers’ behaviour and interests in terms of pregnancy-related topics in developed countries. However, there is little information in understanding the topics searched by first-time fathers in the Southeast Asia region, including Indonesia, where cultural diversity and technological advancements intersect [26, 27]. These situations might influence the information-seeking behaviour of the information seekers [28, 29].

Therefore, this study aims to explore pregnancy-related topics being searched and reasons for the search by first-time Indonesian fathers. Our research questions are outlined as follows: i) What topics related to pregnancy were searched by first-time fathers? and ii) What are the underlying reasons for first-time fathers performing the search related to the pregnancy topics? The results of this study will contribute to the domain knowledge of information-seeking behaviour. Furthermore, it will also add knowledge about the reasons why first-time fathers search for particular topics around pregnancy, as previous studies have not extensively explored this aspect.

## Methods

### Study design

In investigating the information-seeking behaviour of first-time fathers in Indonesia, Wilson’s Information Behaviour Model [30] provides a robust theoretical framework. This model explains the complex process by which individuals identify their information needs, select appropriate channels for seeking information, and navigate barriers in accessing and utilising information effectively. By applying Wilson’s model to this study, the researcher can systematically explore the specific topics searched by fathers during pregnancy and the reasons behind their pursuit of these particular topics.

To enrich this framework, this study employed a grounded theory approach to examine topics searched by first-time expectant Indonesian fathers and the underlying reasons behind the search. Grounded theory methodology allows theories to emerge from the data itself [31]. Given the limited understanding of information-seeking practices among first-time fathers in Indonesia, this approach was chosen to uncover patterns, explore themes, and generate concepts that are grounded in empirical evidence [31]. This iterative process ensures that the theoretical constructs are grounded in the actual experiences and behaviours of the fathers, thereby enhancing the relevance and applicability of Wilson’s model in this specific context. This methodological selection is particularly advantageous as it facilitates a thorough exploration of the nuanced and context-specific behaviours of first-time expectant fathers.

### Research setting

This study focuses on first-time expectant fathers in Indonesia. Indonesia is one of the developing countries in the Southeast Asia region [32]. As the most populous nation in Southeast Asia [33], Indonesia encompasses a wide range of cultural norms, traditions, and varying levels of health and digital literacy [34–36]. The country’s internal diversity provides a nuanced setting for examining how first-time expectant fathers engage in topics related to pregnancy. Indonesia’s cultural, linguistic, and contextual elements mirror the broader diversity within Southeast Asia [33, 37, 38].

### Research participants

Recruitment advertisements were placed in healthcare facilities *(“JIH”* hospital chain—customer service, website, and social media account) and various online social media platforms (*Bidankita* Instagram account, *Forum Ibu Menyusui* Facebook forum). The recruitment period for this study is from November 2022 to July 2023. Ethics approval was obtained from the University’s Human Ethics Committee prior to recruitment. Eligible participants had to meet specific criteria, such as being at least 21 years old, first-time expectant Indonesian fathers, and having searched for pregnancy-related information online. Additionally, individuals were excluded if they were employed in, held formal qualifications, or were pursuing academic credentials in healthcare or related fields to ensure that health expertise and literacy did not skew findings.

### Data collection

Data collection in this study involved the use of questionnaires and semi-structured interviews. Prior to starting the online questionnaires, participants were required to provide informed consent. Consent approval was stored in the database upon completion of the online questionnaire. During the interview, participants were also verbally informed about the consent process, and the interview commenced after the participants had given their consent. Consent obtained during the interviews was recorded and stored in the recording database. Participants were allowed to select to conduct the interview either in Bahasa Indonesia or in English. The researcher is Indonesian, so translation was done if the participants chose to conduct the interview in Bahasa Indonesia. However, research has shown that using the mother tongue in surveys and interviews can lead to more reliable and valid data [39].

The questionnaire is divided into two parts. The first part collected social demographic information such as age, residential area, educational background, and language preferences to provide background information about participants. Part 2 collects information about health literacy levels, and digital literacy levels to provide the literacy level of participants. Participants were to select from one of the following categories: very low, low, average, high, or very high. The results of the questionnaire were used to provide an overview of the participants of this study and will not be used for analysis.

Semi-structured interviews were employed to gather data that facilitated a deep exploration of participants’ thoughts and experiences [40]. The questions for the interview were open-ended and focused on the research questions outlined in the study. Participants were asked about what topics they searched during the pregnancy journey and were also prompted to elaborate on their main reasons behind the decision to search for these topics. Keywords related to topics mentioned by participants during their information-seeking activities were recorded and subsequently coded by the researcher for the "Topics Searched by First-time Fathers" coding list. Additionally, the reasons provided by the fathers will be coded into the “Reasons for Searching Specific Topics” coding list. All interviews were recorded and transcribed verbatim to ensure a robust connection between the data and the researcher [41].

### Data analysis

Data obtained from the interviews were subjected to qualitative thematic analysis. Verbatim transcriptions of the interview audio recordings were conducted to ensure an accurate representation of participants’ responses [41]. The grounded theory approach was utilised alongside the open coding technique to examine participants’ reasons for searching particular topics. Initially, a list of codes was generated through inductive reasoning. These codes were revisited and revised after every third interview. Then, codes were condensed into themes using constant comparative methods. This involved systematically reviewing each response to identify recurring patterns and key themes [42, 43]. Data analysis began immediately after each interview and continued until it captured the main findings and insights from the data. This process enabled a thorough understanding of fathers’ information-seeking practices regarding the reasons behind search actions [31]. To enhance interpretive validity and intercoder reliability, personal reflexivity practices were implemented [44].

## Results

In this study, a total of 40 participants were interviewed as data saturation has been achieved with 40 participants. Typically, data saturation is reached after conducting approximately 15 interviews, with additional interviews totalling one-third of the initial number [45]. Furthermore, another research shows that data saturation can be attained with the participation of 30 individuals [46]. Correspondingly, Patton [47] suggests that thematic saturation can be achieved through 30 interviews. However, previous studies investigating information-seeking behaviour have employed a participant pool of 40 individuals to reach data saturation [48, 49]. Thus, this study’s inclusion of 30 participants with an additional one-third aligns with previous studies investigating information-seeking behaviour in terms of participant numbers required to achieve data saturation.

### Socio-demographic details of participants

The majority of participants fell within the 26 to 30 age bracket (67.5%). Most participants reside in urban areas (52.5%), while 25% live in suburban areas, and the remainder in rural areas (22.5%). Educational backgrounds are varied, with 65% holding Bachelor’s degrees, followed by Master’s degrees (17.5%) and high school diplomas (17.5%). Language preferences for online searches reveal a preference for the national language (Bahasa Indonesia) among the majority (62.5%), as opposed to local dialects such as Javanese, Sundanese, Balinese, or Betawi. Health literacy levels vary among participants, with 60% of participants demonstrating moderate health literacy and 27.5% exhibiting high health literacy. Digital literacy level is also varied, with 50% demonstrating high proficiency and 40% exhibiting moderate digital literacy skills.

### Topics searched by first-time fathers

The results presented in Table 1 highlight a distinct emphasis on certain pregnancy-related topics among first-time fathers. Notably, there was significant interest in topics such as partners’ health, nutrition and diet, prenatal care, and dos and don’ts guidelines during pregnancy as these topics formed the top four topics. The majority of participants, constituting 84%, showed keen interest in partners’ health-related topics. Following, nutrition and diet, as well as prenatal care, garnered considerable attention, with 80% and 76% of fathers respectively seeking information on these topics. Similarly, dos and don’ts during pregnancy attracted substantial engagement, with 70% of fathers expressing interest in seeking this guidance.

**Table 1:** List of topics searched by first-time Indonesian fathers.

Topics	Number of Participants (Percentage)
Partners’ Health-Related Topics	34 (84%)
Nutrition and Diet	32 (80%)
Prenatal Care	31 (76%)
Dos and Don’ts	28 (70%)
Healthcare Professionals	20 (48%)
Mom and Child Trimester Development	20 (48%)
Pregnancy Products Information	20 (48%)
Healthcare Providers	18 (44%)
Cultural Matters	15 (36%)
Labour and Delivery Process and Preparation	10 (24%)
Traditional or Herbal Medicine	8 (20%)
Financial Matters	8 (20%)
Infant Care	8 (20%)
Preparing for Parenthood	7 (16%)
Medical Procedures and Interventions	7 (16%)
Emotional Support for Mothers	5 (12%)
Traditional Practice	4 (8%)
Religion-Related	4 (8%)
Fathers’ role during Pregnancy	4 (8%)
Parenting Classes and Support Groups	2 (4%)

Conversely, certain topics saw notably lower levels of interest. There were only less than 10% of fathers expressed interest in these bottom four topics. Traditional practice (8%), religion-related aspects (8%), fathers’ role during pregnancy (8%), and parenting classes and support groups (4%) emerged as the least discussed areas. This discrepancy indicates differing priorities and preferences among fathers.

### Reasons for searching on specific topics

Table 2 presents themes categorising first-time fathers’ main reasons for performing online searches for information about pregnancy. The themes are to understand the situation and find the solutions (35%), to support their partner (25%), to add new knowledge (15%), to validate and complete the information obtained (10%), to avoid things that are not desirable (5%), to improve confidence in their new role (5%), and to be better prepared (5%). This shows the diverse motivations underlying first-time expectant fathers’ information-seeking behaviours during pregnancy.

**Table 2:** Reasons for performing the search.

Themes	Quotes from Participants
To understand the situation and find the solutions (35%)	“When my wife is experiencing something distressing, I feel compelled to understand its cause, how to alleviate the symptoms or pain, and whether it is normal or something that should raise concern.”
	“If I can understand when something happened with my partner clearly, then I can also help find the solutions.”
To support their partner (25%)	“When I seek information about pregnancy, back in my mind, I do this to support my partner and help her to face any challenges that lie ahead.”
	“Knowing the do’s and don’ts during pregnancy is important for supporting my partner.”
To add new knowledge (15%)	“All these things are new to me, so I feel the need to learn more about them. This will enable me to answer my partner’s questions and make informed decisions when necessary.”
	“I am still new at this, therefore, I have to improve my knowledge about pregnancy as much as I can. Then, I can not only share the information with my partner but also with others.”
To validate and complete the information obtained (10%)	“We received information from my wife’s friend about a beneficial herbal drink for the child. I sought a scientific explanation before considering its implementation for my wife.”
	“I usually receive interesting pregnancy-related information on my social media. However, I still do further searching to validate this information before implementing the suggestions or recommendations.”
To avoid things that are not desirable (5%)	“By knowing comprehensive health information about the mother and unborn child and implementing it, I hope I can avoid any bad things in the future.”
	“We are interested in understanding the local customs and cultural practices related to pregnancy. We aim to avoid any potential social sanctions, as we are not native to the area and wish to align with local traditions.”
To improve confidence in their new role (5%)	“I am not really confident becoming a new father. I tried to improve it by seeking and gathering information about pregnancy. The more topics I searched, the more I know, the more I become confident.”
	“Even though I am still new, my family and friends expect me as a knowledgeable person related to pregnancy. Therefore, to fulfil their expectation, I seek various topics around pregnancy.
To be better prepared (5%)	“It has become my habit to prepare and plan everything in advance, especially when it comes to pregnancy and the health of my partner and child.
	“As a new father, I need to be better prepared not only during this pregnancy but also during delivery and early infant care. This is to ensure everything goes smoothly.”

In the “To understand the situation and find the solutions” theme, fathers expressed a desire to comprehend their partner’s experiences during pregnancy and sought information to address any distressing symptoms or concerns. As most things happening during pregnancy are considered to be new things by fathers, they have shown a proactive approach when their partners experience something during the pregnancy journey. Fathers feel a need to educate themselves about the various knowledge related to pregnancy. By acquiring this knowledge, they equip themselves with the tools necessary to support their partner and navigate any unforeseen circumstances that may arise. Furthermore, fathers want to ensure their partner and unborn child have a good well-being and a smooth pregnancy journey.

In the “To support their partners” theme, fathers highlighted the importance of knowing the dos and don’ts during pregnancy to effectively support their partners. As a husband, fathers demonstrate a sense of responsibility and commitment to provide the necessary care and assistance throughout the pregnancy. Therefore, by understanding all these dos and don’ts matters, fathers feel their roles as a husband and as an expectant father are fulfilled. By seeking information about pregnancy, first-time fathers demonstrate their commitment to being reliable and empathetic partners.

In the “To add new knowledge” theme, fathers underscore the feeling of entering new uncharted territory during the pregnancy journey. Thus, fathers actively seek to acquire new knowledge to help them navigate. By understanding these new topics, first-time fathers empower themselves to make informed decisions, anticipate potential challenges, and actively participate in the pregnancy journey. This reflects their commitment to continuous learning as they transition into their role as fathers.

In the “To validate and complete the information obtained” theme, fathers emphasised the importance of verifying the accuracy and reliability of information received, particularly when it comes to implementing recommendations to their partners. This is a cautious approach to ensure the safety of any advice or suggestions received. Whether receiving guidance from family members, friends, or online sources, fathers exercise critical thinking and cautious to ensure the information obtained aligns with reputable sources and evidence-based practices.

In the “To avoid things that are not desirable” theme, fathers stated that they understand if they may encounter challenges or conflicts throughout the pregnancy journey. Therefore, by actively seeking information to discern what is desirable and beneficial for their partner and unborn child, fathers can avoid potential risks or adverse outcomes. Not only related to the mother’s and unborn child’s health but also related to local customs and cultural practices. Fathers feel that they might receive local sanctions or unintended consequences if they do not align with the local culture or norms during the pregnancy.

In the “To improve confidence in their new role” theme, first-time fathers experience feelings of uncertainty about their capabilities and responsibilities. As expectant fathers, fathers’ self-confidence is very low when it comes to discussing pregnancy. Seeking information about pregnancy serves as a means to boost their confidence and self-assurance in their new roles as expectant fathers. Acquiring knowledge about pregnancy enables them to navigate parenthood with greater ease and confidence.

In the “To be better prepared” theme, fathers mentioned how they anticipate the arrival of a new family member with careful preparation and planning. Seeking information about pregnancy allows fathers to proactively prepare for the upcoming changes and responsibilities, both during pregnancy and beyond. Fathers not only focus on practical preparations such as preparing healthy foods for their partner, setting up the nursery, and purchasing essentials but also emotional preparations such as seeking support networks. By being better prepared, fathers can approach parenthood with confidence and create better decision-making processes.

## Discussion

### Popular topics among first-time fathers

Most of the popular topics among first-time fathers are related to maternal health and pregnancy care. The popularity of these topics among first-time fathers can be attributed to various factors. Firstly, the predominant emphasis on health-related topics among first-time fathers reflects an immediate concern for the health and well-being of both their partners and unborn children [5]. Fathers prioritise topics such as maternal health, nutrition, and prenatal care, indicating a proactive approach to ensuring the health of their family [8, 14, 25]. Furthermore, fathers also actively searched for topics around services providing healthcare professionals and healthcare providers. This result demonstrates fathers’ commitment to seeking comprehensive and reliable information and resources related to health topics. By accomplishing this, fathers aim to support their partners’ physical and emotional well-being and actively engage in the pregnancy journey which may contribute to optimal maternal and child health outcomes.

The results of this study also indicate that fathers primarily focus on finding topics related to their partners’ well-being during the search process. These popular topics reflect societal norms and expectations that place emphasis on mothers’ health during pregnancy. These topics are closely tied to their partners’ situations throughout the pregnancy journey. The emphasis on the mother aligns with traditional gender roles and societal expectations, where the mother is often viewed as the primary figure in pregnancy and childbirth [50, 51]. Additionally, fathers anticipate that prioritising their partners’ well-being will also positively impact their unborn children [52]. Therefore, the results of this study suggest that fathers demonstrate a clear tendency to prioritise the well-being of their partners, including their unborn children, throughout the pregnancy journey.

### Unpopular topics among first-time fathers

Certain topics such as cultural matters, traditional or herbal medicine, and religion-related information receive less attention from first-time fathers. This could be attributed to several reasons. Firstly, there may be a lack of perceived relevance or urgency regarding these topics compared to health-related matters. Additionally, some topics are considered taboo or less essential to discuss openly. Moreover, the availability of reliable information on these topics in online sources might be limited. This leads to lower engagement among fathers.

The lower engagement with topics related to cultural practices, traditions, and religion among first-time fathers suggests a potentially lesser influence of these factors on their information-seeking behaviour during pregnancy. This finding may reflect a shift towards more modern and secular approaches to pregnancy among fathers in Southeast Asia countries [53]. It could indicate that fathers prioritise practical and evidence-based information over traditional or religious beliefs when seeking information about pregnancy. Nevertheless, it is also essential to consider the multifaceted nature of cultural dynamics in Southeast Asia, including Indonesia, when understanding this trend.

Cultural norms and traditions vary widely across Southeast Asia countries and some fathers may still value and adhere to traditional practices and religious beliefs regarding their daily activities, including pregnancy matters [53, 54]. Results of this study indicate that fathers were still searching for cultural matters, such as local cultural guidance on pregnancy, myths surrounding pregnancy, prohibited activities for pregnant mothers or newly pregnant couples, and some rituals that needed to be accomplished. Therefore, the lower search frequency for these topics may not necessarily indicate a complete disinterest but rather a lesser emphasis on seeking information specifically related to cultural, traditional, and religious aspects. Furthermore, the availability and accessibility of online sources pertaining to cultural, traditional, and religious practices also worth to be considered. Fathers may rely more on offline sources, such as family elders, community leaders, or religious advisors, to guide them on these matters rather than find this information online by themselves. This could be due to the complexity and richness of cultural traditions, which may not be adequately captured or addressed in online platforms.

### Comparison between Indonesia and developed countries–Topics

Comparison between topics searched by first-time fathers in Indonesia and developed countries reveals both similarities and differences in topics searched and reasons for seeking particular topics. For instance, Health-related topics, such as maternal health, nutrition, and prenatal care, are consistently popular among fathers across different regions [6, 8, 19, 21]. Furthermore, focusing on their partners is also consistent with the results of previous studies in developed countries [14, 20]. This reflects universal concerns for maternal and child well-being. However, there may be variations to the extent to which fathers prioritise certain topics based on cultural norms, societal expectations, available resources, digital literacy, and access to healthcare services.

While fathers in developed countries also focus on themselves as the subject of their searches [18], fathers in Indonesia exhibit less interest in topics around fathers themselves. By predominantly focusing on their partner as the subject during the search, fathers may perpetuate an unequal distribution of responsibilities and limit their involvement in crucial aspects of pregnancy [52, 55]. Moreover, this approach may overlook fathers’ own information needs and emotional experiences during this transformative period. Consequently, this might lead to feeling marginalised and excluded from the pregnancy journey, despite actively seeking information about pregnancy online.

Fathers in developed countries may have greater access to alternative sources of information beyond traditional practices, such as scientific literature, online forums, and support groups. This could lead fathers in developed countries to focus more on evidence-based topics when it comes to information about pregnancy. However, they may still search for information related to cultural and traditional practices, but the prevalence and significance of these topics could differ due to cultural differences and varying levels of exposure to traditional beliefs and practices. Fathers in Indonesia may exhibit a stronger attachment to cultural, traditional, and religious practices due to the region’s rich cultural heritage and the prevalence of traditional beliefs and customs. This is because they live in societies where cultural traditions play a central role in family life. However, the results of this study show that fathers in Indonesia tend to prefer evidence-based health topics rather than traditional and cultural topics related to health. Even though some first-time fathers are still seeking topics related to culture, tradition, and religion around pregnancy online, they do it just to add knowledge, validate some information, avoid sanctions from societies, and show respect to elders.

### Popular reasons for searching particular topics

The reasons behind first-time fathers’ searchers for specific topics during pregnancy shed light on their diverse motivations and priorities. The popularity of certain themes of reason over others can be attributed to several factors. For instance, themes such as “understanding the situation and finding solutions”, and “supporting their partners”, are prevalent due to fathers’ inherent desire to ensure the health and well-being of their families. These themes align with the paternal role as caregivers and supporters which reflects societal expectations and personal motivations. This underscores fathers’ sense of responsibility, empathy, and commitment towards ensuring their partners’ well-being and navigating any challenges that may arise. Moreover, themes such as “to add new knowledge” and “to validate and complete the information obtained” reflect fathers’ aspirations to enhance their understanding of pregnancy. These themes highlight fathers’ proactive approach to acquiring information and validating information to empower themselves with the knowledge necessary to make better-informed decisions. While fathers in Indonesia focus on assessing and understanding the situations surrounding pregnancy, fathers in developed countries tend to focus on confirming their role in pregnancy [10]. This suggests that fathers in developed countries may possess a greater understanding of maternal health. Therefore, this allows them to also focus on their own involvement during pregnancy.

## Limitations and future directions

Several limitations should be acknowledged when interpreting the findings of this study on topics searched by first-time fathers. Firstly, while the sample size is representative of first-time expectant fathers in Indonesia, it may limit the generalisability of the results to a broader population. Secondly, relying primarily on self-reported data from interviews introduces the possibility of social desirability bias and recall inaccuracies. Thirdly, the study’s focus on Southeast Asian and Indonesian cultural contexts may restrict the applicability of the findings to other cultural settings. Lastly, despite efforts to ensure diversity, the study’s concentration in Indonesia’s geographical region might not fully encompass the country’s regional variations. These limitations should be considered when interpreting the findings. This situation highlights the necessity for future research to address these constraints and refine our comprehension of first-time expectant fathers’ information-seeking practices.

A potential avenue for further exploration concerning topics searched by first-time fathers involves delving into the topics that were unsuccessfully found by first-time fathers in Indonesian and other Southeast Asia countries or topics that need further assistance. The emotional and mental toll that comes with these types of topics is also a potential area for future research. Additionally, longitudinal studies tracking fathers’ information-seeking behaviours throughout different phases of pregnancy could uncover how these practices evolve over time, particularly related to the topics searched.

## Conclusions

In conclusion, the findings of this study provide valuable insights into the information-seeking behaviours of first-time expectant fathers in Indonesia, particularly in the context of topics searched and reasons behind the searches during pregnancy. The study highlights a predominant emphasis among fathers on prioritising their partners’ well-being throughout the pregnancy journey. This reflects traditional gender roles and societal expectations. Moreover, the study underscores fathers’ proactive approach to seeking health-related information. This reflects their commitment to supporting their partners’ physical and emotional well-being and contributing to optimal maternal and child health outcomes. Additionally, the lower engagement with topics related to cultural practices, traditions, and religion suggests a potential shift towards more modern and secular approaches to pregnancy in Indonesia.

On a theoretical level, the results of this study fill the existing research gap and enhance the global understanding of fathers’ involvement in the complex process of pregnancy. This study adds knowledge to existing theories of gender roles, parental involvement, and health information-seeking by explicating specific topics that fathers in Indonesia search for during pregnancy. Moreover, by exploring the reasons behind the searches, this study provides insights into psychological and social motivations driving fathers’ information-seeking behaviour. This contributes to theories related to the father’s roles and involvement in pregnancy and their transition to parenthood.

On a practical level, these findings have implications for healthcare providers, support organisations, and content developers. For example, if fathers commonly search for information about prenatal nutrition, healthcare providers could offer targeted educational materials on this topic. Support organisations could also develop programs tailored to the topics searched by fathers in this study such as offering support groups for expectant fathers who want to support their partners. Content developers could design user-friendly interfaces and customisable content that can meet their information needs.

Overall, this study contributes to bridging the gap between theoretical advancements and practical applications in the context of topics searched by first-time fathers and the reasons behind their searches around pregnancy in Indonesia. By addressing cultural nuances and contextual factors, it provides actionable insights for promoting paternal involvement and fostering supportive pregnancy environments. This ultimately can contribute to better maternal and child health outcomes.

## Supporting information

S1 File List of topics searched by first time fathers. (PDF)

S2 File Transcript of reasons by first time fathers. (PDF)
