# Supplementary material for: Topics searched by first-time Indonesian fathers during pregnancy journey: An exploratory study
Source: PLoS One. 2024 Jul 26;19(7):e0307051. doi: 10.1371/journal.pone.0307051 (PMC11280144; doi:10.1371/journal.pone.0307051)
Supplement: S1 File — (PDF) [file pone.0307051.s001.pdf]

Topics	Number of Participants (Percentage)	DS733	RN709	AX966	AW021	IF354	FA098	HY100
Partners' Health-Related Topics	34 (84%)	Nausea	Cramped	Spotting		Backache	Swollen Body Parts	Hormonal Things
Nutrition and Diet	32 (80%)	Recommended Food	Food to avoid	Food to avoid	Food to avoid	Food proportion	Recommended food	Amount of vitamin
Prenatal Care	31 (76%)	Activities	Exercies		ANC schedule		sexual activities	Exercies
Dos and Don'ts	28 (70%)	Do's and don't's		Do's and don't's	Do's and don't's	What to avoid	What to avoid	What to avoid
Healthcare Professionals	20 (48%)				Obgyn Doctor	Obgyn Doctor	Obgyn Doctor	
Mom and Child Trimester Development	20 (48%)	Unborn Child Dev Stages			Trimester Cycle			
Pregnancy Products and Services Information	20 (48%)					Milk		
Healthcare Providers	18 (44%)			Service Provided by Hospital	Hospital Service			Hospital Service
Cultural Matters	15 (36%)	Local Culture		Local Culture			Things not allowed to do	
Labour and Delivery Process and Preparation	10 (24%)		labor process	preparation		labor process		
Traditional or Herbal Medicine	8 (20%)	coconut water				green beans		

Financial Matters	8 (20%)	birthing cost			antenatal care cost	national health insurance coverage	birthing cost	
Infant Care	8 (20%)				take care newborn baby			
Preparing for Parenthood	7 (16%)		how to raise kids				parenting	
Medical Procedures and Interventions	7 (16%)							
Emotional Support for Mothers	5 (12%)							
Traditional Practice	4 (8%)							massage
Religion-Related	4 (8%)							
Fathers’ role during Pregnancy	4 (8%)							
Parenting Classes and Support Groups	2 (4%)							

GS089	HR817	AR396	AZ032	AR635	SR661	EM325	BA823	AZ023	AD923	PB735
Headache	Vomiitting	Partner's health	Nose-bleeding	Partner's Well being	Headache	High Fever	Swollen Body Parts	Cramped	Hormonal Things	supporting partners' health
Nutrition needed	Menu for pregnant women		Allergies	Food to avoid	Food proportion	Recommended food		Food to avoid	Dietary requirement	Fresh food
		Exercies	Activities	Activities	Activities	Activities	Activities	Activities	ANC schedule	Pregnancy Screening
Pregnancy Precautions	Do's and don't's	Do's and don't's	What to avoid	Safe practices	What to avoid	What to avoid	Pregnancy Precautions	Pregnancy Precautions	What to avoid	Do's and don't's
	Obgyn Doctor	Obgyn Doctor		Doctor Pro Normal Birth	Doctor's reputation	Midwive service	Obgyn Doctor		Obgyn Doctor	Obgyn Doctor
Pregnancy Stages		Trimester Cycle	Pregnancy Stages			Pregnancy Stages			Unborn Child Dev Stages	Pregnancy Stages
Vitamin			Massager	Leg warmer	Gym Ball				Girdle	Milk
Hospital Near Me	Hospital Cooperate with Insurance		Hospital Service		Service Provided by Hospital		Hospital Service		Hospital Service	Hospital Cooperate with Insurance
		Things not allowed to do						Local Culture		
signs of delivery		false contraction		labor process			tips for safe labor	amniotic fluid		
		coconut water		turmeric water		coconut water		certain egg		

						USG cost				birthing cost
take care newborn baby			vaccines						baby products	
	parenting				parenting		parenting			parenting
					how to support partners conditions			partners' emotional changes		
			massage							

RO945	DK028	UC095	EN017	RN026	HA175	MU234	DU022	ZA100	AM341	SP053
Sore	Cramped	Stiff	Red mark in stomach	Hormonal Things	Nausea	Headache		Vomitting		Itchy
Seafood	Food to avoid		Food to avoid	Vitamin Needed	Vitamin Needed	Food to avoid	Allergies	Food to avoid	Food to avoid	Food proportion
Prenatal checkup	ANC Schedule	Exercices	Activities	Activities	Activities	Activities	Activities	Activities	Activities	
Do's and don’t's	Do's and don’t's	Do's and don’t's	Do's and don’t's		What to avoid	Do's and don’t's		Do's and don’t's	What to avoid	What to avoid
		Obgyn Doctor	Obgyn Doctor	Obgyn Doctor		Midwive service	Obgyn Doctor			Obgyn Doctor
	Pregnancy Stages	Unborn Child Dev Stages	Physical Changes		Pregnancy Stages	Physical Changes			Physical Changes	Physical Changes
Maternity Products and Reviews			Baby Essential	Maternity Products and Reviews	Pregnancy Gear	Hypnobirthing Training			Milk	Maternity Products and Reviews
Hospital Cooperate with Insurance	Hospital Service			Hospital Service		Hospital Service	Hospital Service			
Myth			Local Culture				Things not allowed to do		Things not allowed to do	Things not allowed to do
	hypnobirthing	preparation								
	spinach									

	birthing cost			birthing cost						
					bathing baby			baby massage		
			good school							
		Caesar preparation					pregnancy test		blood test	
	how to support partners conditions						recognise partner's stress			
		Fathers' involvement				Role advice				fathers' responsibilities

IR724	AF093	PG431	DA653	KR157	RN003	AR388	RA810	BA112	TM811	FA695
Nausea			Nausea	Migraine	Morning sick	Cramped	Sore		Vomitting	Nausea
Nutrition needed	Food to avoid	Food to avoid	Food proportion	Food to avoid	Food to avoid					
Screening			sexual activities			Activities	Activities	Activities	Activities	Activities
Safe practices		What to avoid								
Midwive service			Doctor's reputation				Doctor's reputation			
			Pregnancy Stages	Pregnancy Stages	Pregnancy Stages		Physical Changes	Trimester Cycle		
Pillow and Bolster			Calming music		Essential oil		Pregnancy Gear	Milk		Milk
Service Provided by Hospital						Hospital Service				Hospital Near Me
	Myth			Local Culture		Ritual	Local culture		Local culture	
									turmeric water	

	take care newborn baby			take care newborn baby						
					pregnancy test		pregnancy test	pregnancy test		pre-birth test
					partners' behaviour					
massage		therapy for easing delivery								
	where to go for praying (pilgrimage)	prayers	prayers	prayers						
						fathers' responsibilities				
								parent class or support groups	parent class or support groups	
