# Supplementary material for: Topics searched by first-time Indonesian fathers during pregnancy journey: An exploratory study
Source: PLoS One. 2024 Jul 26;19(7):e0307051. doi: 10.1371/journal.pone.0307051 (PMC11280144; doi:10.1371/journal.pone.0307051)
Supplement: S2 File — (PDF) [file pone.0307051.s002.pdf]

Themes	Total	Participants	Quote From Participants
To understand the situation and find the solutions	14	KR157	"When my wife is experiencing something distressing, I feel compelled to understand its cause, how to alleviate the symptoms or pain, and whether it is normal or something that should raise concern."
		DS733	"If I can understand when something happened with my partner clearly, then I can also help find the solutions."
		RO945	"When we first found out about the pregnancy, I wanted to learn all I could about prenatal care to ensure we were following the best practices for a healthy pregnancy. This included understanding the complexities of our local healthcare system and knowing what steps to take at each stage."
		AZ023	"Nutrition is crucial during pregnancy, so I focused on my partner's diet. It was important to learn how to incorporate our local cuisine into a balanced diet and avoid foods that could be harmful. Understanding these details helped us make better dietary choices."
		RN709	"Staying informed about partners' health topics was vital to ensuring both of us remained healthy throughout the pregnancy. This included looking into local environmental factors and how they might affect us, so I could better support my partner's health."
		RN026	"Knowing the dos and don'ts during pregnancy was necessary to avoid potential risks. This meant looking into both local beliefs and medical advice to understand what we should and shouldn't do during the pregnancy."
		IR724	"Finding the right healthcare professionals in our area was a top priority. Researching their credentials and specialties helped us make an informed decision about who would provide the best care for my partner, given the variability in access to specialized care."
		AZ032	"I was constantly curious about our baby's development. Learning about the different stages of fetal growth each trimester helped me understand what my partner and baby were going through, which was very reassuring."
		MU234	"Choosing the right pregnancy products can be overwhelming, so I researched extensively to determine which ones were necessary and culturally appropriate. This helped us avoid unnecessary expenses and ensure we had the best items for my partner."
		AF093	"Exploring the healthcare providers available to us was crucial. We needed to know who could offer the best care, so I looked into different hospitals and clinics to compare their services and facilities."

		AR635	"Understanding cultural practices around pregnancy was important to me. I wanted to see how traditional practices could be integrated into our journey while ensuring we followed modern medical advice."
		RN003	"Preparing for the labor and delivery process was a big part of my research. Knowing what to expect and how to support my partner during this critical time helped ease our anxiety and made us feel more prepared."
		PB735	"The financial aspects of having a baby were also on my mind. I looked for advice on managing pregnancy-related expenses and budgeting for the baby's arrival to ensure we were financially ready."
		HA175	"Traditional and herbal medicine is common in our culture, so I wanted to find safe remedies that could help my partner with pregnancy symptoms. This involved researching which herbs were safe and beneficial."
To support their partner	10	AR396	“When I seek information about pregnancy, back in my mind, I do this to support my partner and help her to face any challenges that lie ahead.”
		SR661	“Knowing the do’s and don'ts during pregnancy is important for supporting my partner.”
		BA823	"Researching the best prenatal vitamins was a top priority for me. I wanted to ensure my partner got all the necessary nutrients, so I spent time comparing different supplements and reading reviews to find the best option."
		UC095	"Helping my partner with morning sickness was important. I searched for remedies and tips to offer her some relief, whether it was through dietary changes or other natural methods."
		AM341	"Understanding her cravings and aversions supported her dietary needs better. I learned about why certain foods were beneficial or harmful during pregnancy and how to accommodate her changing tastes."
		AR388	"Realizing the importance of rest for my partner, I looked for ways to help her relax and get enough sleep. This included researching comfortable sleeping positions and creating a restful environment at home."
		IF354	"Providing emotional support was crucial during her pregnancy. I searched for tips on how to be there for her, listen to her concerns, and offer reassurance whenever she felt anxious or stressed."
		HY100	"Staying active is important during pregnancy, so I looked up safe exercises my partner could do. This helped us incorporate physical activity into her routine without risking her health."

		DU022	"Assisting with her prenatal exercises was a key focus for me. I wanted to know which exercises were safe and beneficial, so I could support her in staying healthy and fit throughout the pregnancy."
		DK028	"Understanding my role during labor and delivery was essential. I researched how I could best support her during this time, whether it was through physical presence, encouraging words, or practical help."
To add new knowledge	6	FA695	“All these things are new to me, so I feel the need to learn more about them. This will enable me to answer my partner's questions and make informed decisions when necessary.”
		AX966	“I am still new at this, therefore, I have to improve my knowledge about pregnancy as much as I can. Then, I can not only share the information with my partner but also with others.”
		ZA100	"Gaining more knowledge about the stages of fetal development was important to me. I wanted to understand how our baby was growing and what changes to expect each month, which made the experience even more exciting."
		RA810	"Reading about the benefits of prenatal yoga was enlightening. I discovered how it could help my partner stay fit, reduce stress, and prepare her body for labor, so we decided to incorporate it into her routine."
		FA098	"Exploring different birthing methods helped us understand our options. We learned about natural births, C-sections, and other methods, which allowed us to make an informed decision about our preferred birth plan."
		GS089	"Learning about how pregnancy affects a woman's body was fascinating. It gave me a deeper appreciation for what my partner was going through and helped me support her better."
To validate the information obtained	4	SP053	“We received information from my wife's friend about a beneficial herbal drink for the child. I sought a scientific explanation before considering its implementation for my wife.”
		EM325	“I usually receive interesting pregnancy-related information on my social media. However, I still do further searching to validate this information before implementing the suggestions or recommendations.”
		BA112	"Looking up multiple sources confirmed the safety of certain foods during pregnancy. I needed to make sure the dietary advice we received was consistent and reliable."
		AW021	"Verifying information on pregnancy supplements was important. I wanted to ensure my partner was taking the best possible vitamins and minerals, so I cross-referenced different recommendations."

To avoid things that are not desirable	2	EN017	“By knowing comprehensive health information about the mother and unborn child and implementing it, I hope I can avoid any bad things in the future. ”
		TM811	“We are interested in understanding the local customs and cultural practices related to pregnancy. We aim to avoid any potential social sanctions, as we are not native to the area and wish to align with local traditions.”
To improve confidence in their new role	2	HR817	“I am not really confident becoming a new father. I tried to improve it by seeking and gathering information about pregnancy. The more topics I searched, the more I know, the more I become confident.”
		AD923	“Even though I am still new, my family and friends expect me as a knowledgeable person related to pregnancy. Therefore, to fulfil their expectation, I seek various topics around pregnancy.
To be better prepared	2	PG431	“It has become my habit to prepare and plan everything in advance, especially when it comes to pregnancy and the health of my partner and child.
		DA653	“As a new father, I need to be better prepared not only during this pregnancy but also during delivery and early infant care. This is to ensure everything goes smoothly. ”
